# Hepatic portal venous gas after colonic endoscopic submucosal dissection: a case report

**DOI:** 10.1016/j.igie.2023.07.009

**Published:** 2023-07-20

**Authors:** Yasuyuki Tanaka, Kentaro Aoki, Keijiro Okada, Shigehiko Fujii

**Affiliations:** Department of Gastroenterology and Hepatology, Kyoto Katsura Hospital, Kyoto, Japan

Colonic endoscopic submucosal dissection (ESD) is widely used as a minimally invasive therapeutic procedure for colorectal tumors. These procedures could lead to a risk of adverse events such as bleeding and perforation. Hepatic portal venous gas (HPVG) is an extremely rare adverse event associated with colonic ESD; there have been only 2 reported cases of this adverse event.^1^^,^^2^

A 74-year-old woman was referred to our hospital for treatment of early colon cancer (25 mm in diameter) in the transverse colon (Fig. 1A). ESD with carbon dioxide insufflation was completed, with no apparent adverse events (Fig. 1B and 1C). The procedure took a relatively long time (178 minutes) due to mild fibrosis of the submucosa and because it was performed by a nonexperienced endoscopist under the supervision of an experienced endoscopist. Five hours after the procedure, the patient developed sudden high fever, chills, and hypotension (systolic blood pressure <80 mm Hg) with mild abdominal pain. Laboratory data showed an increased white blood cell count of 17,900/μL. Abdominal CT imaging revealed HPVG extending throughout the superior and inferior mesenteric vein (Fig. 2A and B), and the transverse colon was edematous (Fig. 2C). However, no free air or ascites was observed. Considering the possibility of bacteremia, the patient was managed conservatively with fasting and intravenous antibiotic treatment. The body temperature and white blood cell count normalized within a few days, and the patient was discharged on post-ESD day 7. Two bottles of blood culture were obtained at the time of the fever, and tests of both eventually yielded negative results.

**Figure 1:**
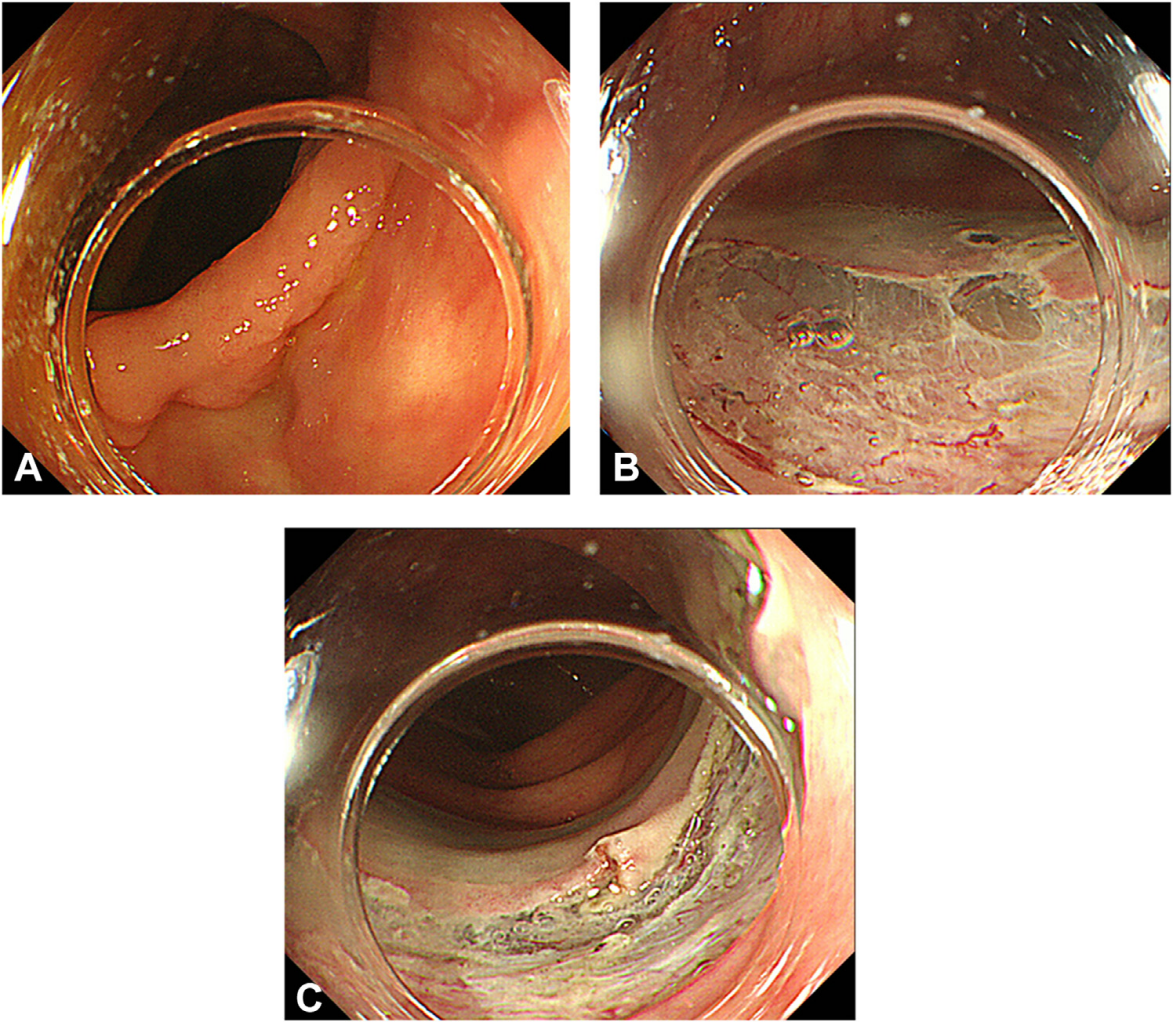
**A,** EGD showing a 25 mm type 0 to IIa lesion in the transverse colon. **B and C,** Appearance of the iatrogenic ulcer after endoscopic submucosal dissection with no apparent adverse events.

**Figure 2:**
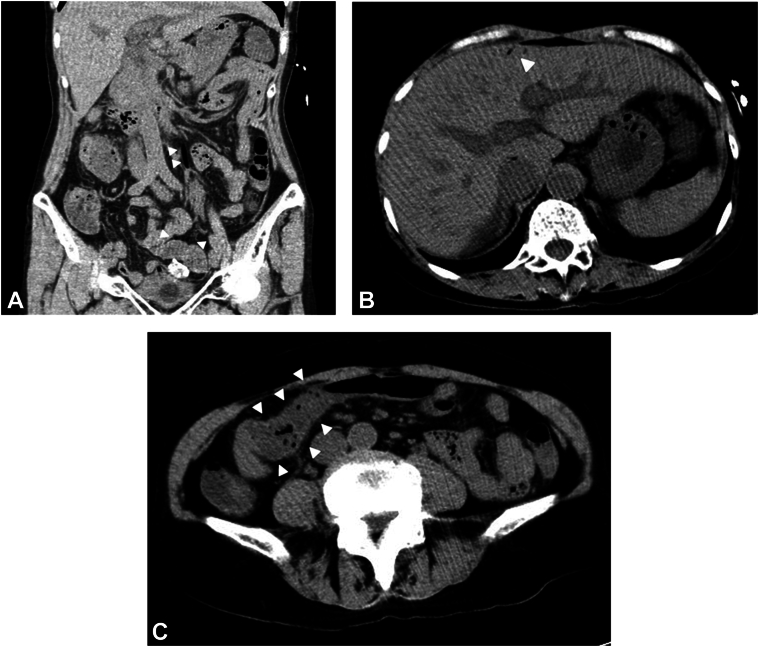
**A and B,** CT imaging 5 hours after endoscopic submucosal dissection revealed gas in the peripheral branches of the liver and the mesenteric veins (*white arrows*), indicating hepatic portal venous gas extending throughout the superior and inferior mesenteric vein. **C,** The transverse colon was edematous, but no free air or ascites was observed.

The mechanism for HPVG remains unclear, but intracorporeal factors such as mucosal damage and bowel distension may be responsible for the development of HPVG.^3^ In addition, cases of iatrogenic HPVG have been reported, especially in patients with inflammatory bowel disease after colonoscopy.^4^^,^^5^ In our case, we speculated that a prolonged procedure with extensive insufflation permitted bowel gas to access the portal venous circulation through the iatrogenic ulcer, as no other etiologic feature of HPVG was noted and the onset occurred a short time after this procedure.

There have been only 2 reported cases of this adverse event associated with colonic ESD^1^^,^^2^ and, in addition to our case, all cases were able to be treated conservatively. Endoscopists performing colorectal ESD, especially for prolonged procedures, should be cognizant of HPVG, a rare adverse event with a unique radiologic sign, which can be effectively managed with conservative treatment.

## Patient Consent

The authors have received appropriate patient consent for the publication of this article.

## Disclosure

All authors disclosed no financial relationships.
